# Gaze detection as a social cue to initiate natural human-robot collaboration in an assembly task

**DOI:** 10.3389/frobt.2024.1394379

**Published:** 2024-07-17

**Authors:** Matteo Lavit Nicora, Pooja Prajod, Marta Mondellini, Giovanni Tauro, Rocco Vertechy, Elisabeth André, Matteo Malosio

**Affiliations:** ^1^ Institute of Intelligent Industrial Technologies and Systems for Advanced Manufacturing, National Research Council of Italy, Lecco, Italy; ^2^ Industrial Engineering Department, University of Bologna, Bologna, Italy; ^3^ Human-Centered Artificial Intelligence, University of Augsburg, Augsburg, Germany; ^4^ Catholic University of the Sacred Heart, Psychology Department, Milan, Italy

**Keywords:** human-robot interaction, industry 5.0, gaze estimation, natural behavior, human-centered computing

## Abstract

**Introduction:** In this work we explore a potential approach to improve human-robot collaboration experience by adapting cobot behavior based on natural cues from the operator.

**Methods:** Inspired by the literature on human-human interactions, we conducted a wizard-of-oz study to examine whether a gaze towards the cobot can serve as a trigger for initiating joint activities in collaborative sessions. In this study, 37 participants engaged in an assembly task while their gaze behavior was analyzed. We employed a gaze-based attention recognition model to identify when the participants look at the cobot.

**Results:** Our results indicate that in most cases (83.74%), the joint activity is preceded by a gaze towards the cobot. Furthermore, during the entire assembly cycle, the participants tend to look at the cobot mostly around the time of the joint activity. Given the above results, a fully integrated system triggering joint action only when the gaze is directed towards the cobot was piloted with 10 volunteers, of which one characterized by high-functioning Autism Spectrum Disorder. Even though they had never interacted with the robot and did not know about the gaze-based triggering system, most of them successfully collaborated with the cobot and reported a smooth and natural interaction experience.

**Discussion:** To the best of our knowledge, this is the first study to analyze the natural gaze behavior of participants working on a joint activity with a robot during a collaborative assembly task and to attempt the full integration of an automated gaze-based triggering system.

## 1 Introduction

With the rise of the concept of Industry 4.0 and the resulting widespread adoption of cobots, the working conditions are changing rapidly (Weiss et al., 2021). In this prolific environment, research strives to move away from technology-driven approaches towards a value-driven era that, besides efficiency, focuses on the workers’ wellbeing and involvement (Schneiders and Papachristos, 2022), the so called the “fifth industrial revolution” (Xu et al., 2021). Therefore, there is a growing need to study the experience of operators who are now working with cobots in order to increase their wellbeing and reduce the risk of social isolation (Nicora et al., 2021).

One of the crucial aspects of designing a human-robot collaborative (HRC) production system is the tuning of the assigned workload since it can significantly impact the operator’s wellbeing. For example, a high workload is associated with distress, high blood pressure, and other indicators of low wellbeing (Ilies et al., 2010). On the other hand, boredom at work leads to distress and counterproductive work behavior (van Hooff and van Hooft, 2014). Both this scenarios are possible when working together with an automatic system which is intrinsically blind to how the operator subjectively perceives the workload throughout his/her shift. Due to these considerations, it is important to adapt the production rhythm to the level of productivity of the operators. Another aspect greatly impacting the wellbeing of operators is the experience of social isolation when working inside a robotic productive work cell where the usual human colleague is substituted by an automatic system. In non-industrial settings, for instance in hospitals or elderly care, studies show that specifically designed robotic solutions can be effective in reducing social isolation (Sarabia et al., 2018). Extending this concept to the industrial context, a cobot capable of interacting with the operator in a natural and social manner may be effective in reducing social isolation. To achieve such a goal, human-robot collaboration strategies should be inspired by everyday human-human interactions, which rely on a variety of perceptual cues (Hadar et al., 1983; Bull and Connelly, 1985; Argyle et al., 1994). For instance, individuals instinctively direct their gaze towards their intended collaborators before initiating collaborative activities (Cary, 1978). If such behavior can be elicited during interactions with cobots, gaze direction can serve as a natural cue to communicate the intention to collaborate.

In fact, such a solution holds promise for real-time adaptation of the production rhythm to the user while, at the same time, providing social experiences akin to working with a human colleague. To this end, we perform an analysis of the natural gaze behavior of participants collaborating with a cobot in an assembly task (Experiment 1). A novel aspect of our study is the joint activity setup, where the human and the robot manipulate the object together. Previous studies (Huang and Mutlu, 2016; Shi et al., 2021) have investigated gaze behavior for industrial applications, however, the task usually involves either the human or the robot picking an object, but not lifting it together. Moreover, after demonstrating the feasibility of using automatic attention recognition in industrial collaborative scenarios as a trigger for initiating joint activity, we pilot the fully integrated system to collect quantitative data and subjective comments over the augmented interaction experience (Experiment 2).

## 2 Background and related works

### 2.1 Gaze in human-human interactions

Gaze is one of the communicative signals used from birth, and the number of scientific studies in this regard is really high. Gazing at a person or an object is an apparently simple act that implies at first the ability to synchronize the movements of the eyes, head, and body. With cognitive development, infants start to use intentional communication (Camaioni, 1992), and eye contact becomes a common precursor to initiating joint attention, namely, the shared focus of two individuals on an object (Hamilton, 2016). In this regard, Cary (1978) underlined that direct eye-gaze displays the willingness to interact; in particular, he watched videos of 80 students who did not know each other, inside a waiting room. What emerged was that when two people started a conversation, this was almost always preceded by eye contact. Ferri et al. (2011) conducted a series of experiments in which a subject grasped food from the table in front of him and placed it in the mouth of a person sitting on the other side. They found that the direct gaze of the person in front influences the performance of the gesture, proposing that the gaze makes a social request effective (to be fed) by activating a social affordance. Innocenti et al. (2012) studied the impact of gaze on a requesting gesture (i.e., grabbing an empty glass with the implicit request to fill it). The study demonstrated that the mere presence of a direct gaze induced a measurable effect on the subject’s response in the initial phase of the sequence. Some authors have also studied the effect of direct gaze on neural correlates. In an examination of several theories regarding the eye contact effect, Senju and Johnson (2009) propose that perceived eye contact is initially detected by a subcortical route that modulates the activation of the social brain. Therefore, eye contact is closely linked to social actions not only from a behavioral point of view but also from a biological point of view.

### 2.2 Gaze in robotics

The analysis of gaze has already been used in the past to enhance the interaction of humans and robotic systems. Often, gazing capabilities have been implemented within humanoid robots in order to expand on their social appearance (Admoni and Scassellati, 2017) and to make them more predictable in their collaborative actions (Boucher et al., 2012). However, this study focuses on the analysis of the natural gaze behavior of human participants in industrial HRC scenarios.

The role of gaze in HRC was first studied using humanoid social robots in puzzle scenarios. Mehlmann et al. (2014) showed that a robot able to follow the user’s referential gaze sped up a collaborative sorting task, reduced the number of placement attempts, and required fewer clarifications to resolve misconceptions. Palinko et al. (2016) studied the effectiveness of gaze information in facilitating a collaborative task. A specific gaze sequence inspired by joint attention in human-human interaction triggered the robot’s behavior. The participants were not instructed on what would activate a particular behavior of the robot that was required to complete the task. The participants tried various communication techniques like talking, pointing, etc., and eventually succeeded in the task. Since the participants succeeded in the task without explicit knowledge of how to activate the robot, the gaze-based interaction was deemed natural.

Recently, studies have also started considering industrial robots which are typically robotic arms. This distinction in terms of the type of robot is crucial because humans may behave differently when there is a human-like face. Huang and Mutlu (2016) designed a setup where the robot picked the pieces selected by the user. The selection was voiced by the user. They demonstrated that collaboration performance improves when the robot can anticipate the user’s choice based on their gaze behavior. Shi et al. (2021) used a similar setup to demonstrate how to recognize the user’s intention to pick an object solely based on their gaze behavior. Saran et al. (2018) trained a deep-learning model to track the user’s gaze from the perspective of the robot and demonstrated that it is possible to determine whether the user’s attention is directed towards an object or the robot in real-time without dedicated eye trackers. Their study did not involve any collaborative task.

In most of the existing studies, gaze behavior serves a functional role (e.g., communicating a choice) and is often required to complete the task. Moreover, the emphasis is typically on performance (e.g., faster completion, lower number of trials). In our study, we explore gaze as a social cue that naturally occurs during industrial human-robot collaboration.

According to Christiernin (2017), there are three levels of collaboration: Idle Robot (Level 1), Human as Guide (Level 2), and Cooperation/Full Interaction (Level 3). Previous works mentioned in the literature typically fall into Level 1 or 2, resulting in imbalanced collaboration, where one partner predominantly handles the task. This limitation affects aspects like waiting for the other partner to complete their actions, synchronizing with the partner, etc., and the associated gaze behavior. Our study utilizes a setup that can be classified as a Level 3 collaboration and involves joint manipulation of the assembly object. To the best of our knowledge, this is the first study that attempts to analyze and exploit the natural gaze behavior of participants performing a joint activity with a cobot during an industrial task.

## 3 Materials and methods

### 3.1 Experimental setup

**FIGURE 1:**
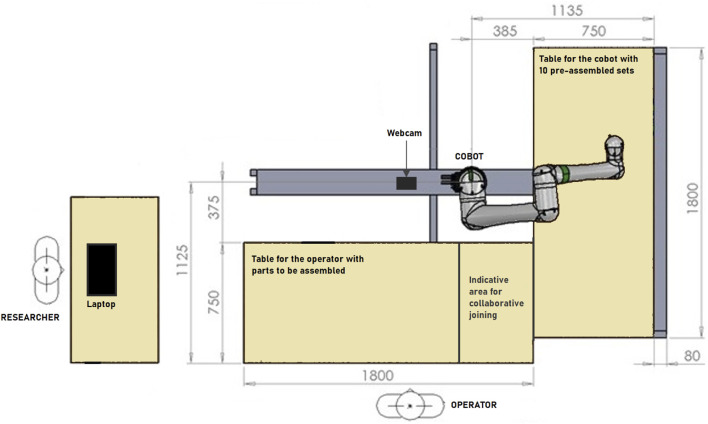
Schematic top-view of the experimental workcell.

**FIGURE 2:**
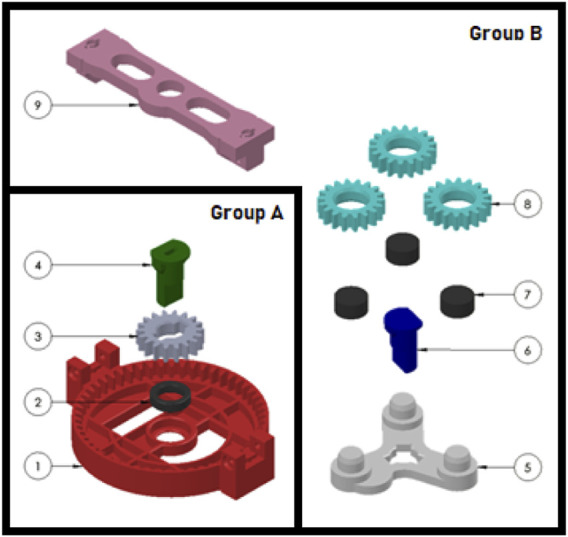
Pre-assembled components for the cobot (Group **(A)**) and components assigned to the operator (Group **(B)**).

**FIGURE 3:**
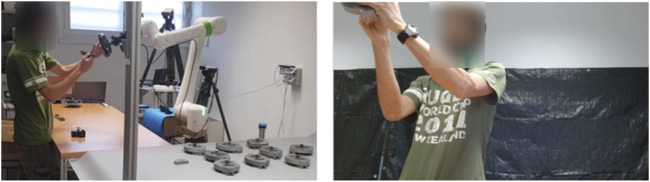
The images show the joint activity between the cobot and the participant from two different viewpoints. On the left is an overview of the setup from the side. On the right is a frame taken from the front camera recordings that were used in the analysis.

A collaborative industrial scenario was recreated in a lab environment. The setup was made up of two distinct areas where the cobot and the operator work on their own sub-assemblies and a common area for collaborative joining to happen. With reference to Figure 1, an L-shaped formation was used to create said zones, plus a separate workstation for the researcher to monitor the system. The selected cobot was a Fanuc CRX10ia/L equipped with a Robotiq Hand-e parallel gripper for pick and place operations. Also, a Logitech Pro C920 HD webcam was placed on the support structure of the cobot, around 1.5 m away from the operator. As a common industrial application and complex assembly scenario, we took inspiration from Li et al. (2021) and selected a 3D-printed planetary gearbox (Redaelli et al., 2021) as a product to be assembled collaboratively by the operator and the cobot. With reference to Figure 2, four components were assigned to the cobot (Group A), and the remaining five components were instead to be assembled by the operator (Group B). A typical assembly cycle involved two phases: the assembly phase and the joint activity. During the assembly phase, the operator gathered the components belonging to Group B and assembled them together. First, a shaft (label 6) was inserted in the carrier (label 5). Then, three bearings (label 7) were placed on the shafts of the carrier and three satellite gears (label 8) were mounted on the bearings. The joint activity followed, with the cobot and the operator meshing the gears of their respective sub-assemblies together to produce the completed gearbox. In order to have more freedom in the timing at which the robot is ready to bring the sub-assembly towards the user, the components of Group A were pre-assembled and ten of these sets were placed on the table of the cobot, ready to be picked up as shown in Figure 3.

### 3.2 Tools

**FIGURE 4:**
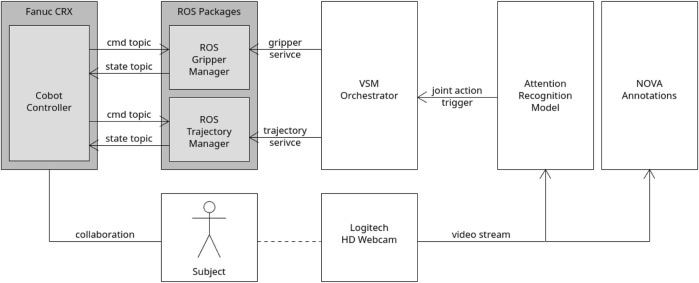
Schematic architecture of the interaction between the different software modules.

Figure 4 depicts a simplified scheme of the software architecture and of the way they are interfaced to each other. On top of that, the following subsections report a description of each one of them in more detail.

#### 3.2.1 Attention recognition model

While designing the setup, we identified three main areas of interest in the environment: the cobot, the table (looking at the table while assembling), and anywhere else (looking at the clock, window, etc.). Consequently, we trained a deep learning model that takes face images as input and classifies the gaze direction into these three areas. In the training process, we employed a transfer learning technique, where the weights of a gaze estimation model were leveraged for training the attention recognition model.

First, we trained a convolutional neural network (VGG16 architecture) using ETH-XGaze face image dataset (Zhang et al., 2020; Ghosh et al., 2023). This model estimates the gaze direction in terms of pitch and yaw. Then, we fine-tuned the prediction layers of this model to map the gaze to one of the areas of interest (three classes). To fine-tune the model, we collected images from volunteers in a guided gaze setting using the same setup as the current study. This fine-tuned model achieved an accuracy = 94.3% and an f1-score = 94%.

We validated the model in a non-guided setting, 81.6% and an f1-score = 81.8%. Upon manual inspection of the prediction results, it was found that the drop in performance was predominantly due to the misclassification of distracted samples as gaze at table. This was because, in the non-guided setting, some of the participants got distracted by objects on the table. However, the model was robust in predicting non-guided gazes towards the cobot (around 90% recall), which is the label primarily utilized in our analysis. The details about the training procedure and validation of this model can be found in Prajod et al. (2023). The mentioned model is the one utilized for both Experiment 1 and Experiment 2 presented in this paper.

#### 3.2.2 NOVA annotation

The participants performed two primary activities: assembling their own sub-assembly and joining the sub-assemblies along with the cobot (joint activity). During both experiments, videos of the interactions were recorded and then annotated using the NOVA tool (Baur et al., 2013), which also allowed us to visualize the predictions from the attention recognition model as a stream. Depending on the goal of each experiment, a specific annotation logic was adopted.

• Regarding Experiment 1, we focused on the gaze behavior of the participants, especially the few seconds leading up to the joint activity. Therefore, for each assembly cycle, we annotated the frame where the cobot arrives for the joint activity.
• Regarding Experiment 2, we wanted to quantify how successful the collaboration between the participants and the fully integrated system was. Therefore, for each assembly cycle, we annotated the frame when the participant is done with his/her part of the assembly and the frame when the cobot receives the trigger and starts moving towards its subassembly.

#### 3.2.3 Visual SceneMaker (VSM)

**FIGURE 5:**
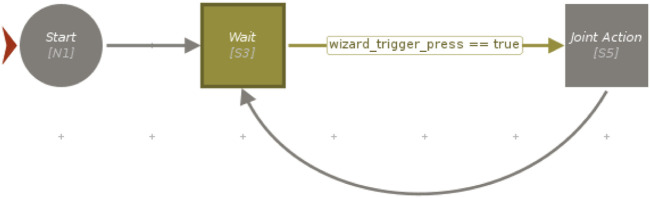
A simplified schema of the VSM project of Experiment 1, iteratively commanding the robot to wait for the wizard trigger before performing the joint action.

**FIGURE 6:**
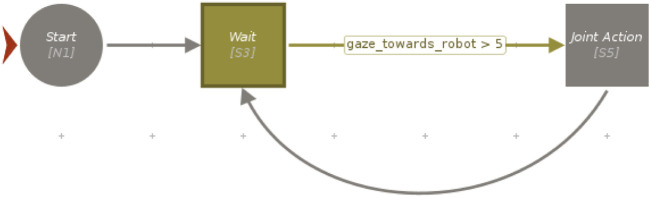
A simplified schema of the VSM project of Experiment 2, iteratively commanding the robot to wait for a gaze longer than 5 frames before performing the joint action.

The high-level state machine needed to orchestrate the task and the logic designed for the two experiments was realized using Visual SceneMaker (VSM) (Gebhard et al., 2012). First, a rosjava-based plugin was coded to enable VSM to communicate with the ROS master through topics and services. After that, the whole assembly task was programmed using VSM functionalities, including the management of the triggers used for the two experiments. In the case of Experiment 1, VSM was simply in charge of listening to a specific keyboard press before commanding the robot to move for joint action, as shown in a simplified form in Figure 5. The change required for Experiment 2 is instead represented in Figure 6, where a more complex integration was required (further details are reported in Section 4.2.2).

#### 3.2.4 Robot operating system (ROS)

In order to control the robot from an external program and not directly from the teach pendant, a software module was developed to interface the controller of a Fanuc CRX10iA/L cobot with ROS Noetic (Stanford Artificial Intelligence Laboratory et al., 2018). This integration was realized using the User Socket Messaging and Remote Motion Interface packages offered by Fanuc in order to create a communication pipeline between the robot and an external computer and to exchange semi-formed control commands. On top of that, the capabilities of RosControl (Chitta et al., 2017) and MoveIt (Coleman et al., 2014) were leveraged to integrate planning and execution functionalities. Finally, a plugin to port the commands coming from VSM into ROS in the form of services was developed making the resulting ROS package a sort of manager connecting the high-level (VSM) and low-level (cobot controller) modules of the system.

### 3.3 Ethical approval

The study has been conducted according to the guidelines of the Declaration of Helsinki and approved by the ethics Committee of I.R.C.C.S. Eugenio Medea (protocol code N. 19/20-CE of 20 April 2020). All the participants were briefed about the study and the details of data treatment before signing an informed consent form.

## 4 Results

### 4.1 Experiment 1–Gaze behavior exploration

Experiment 1 aimed to analyze the natural behavior of users directly collaborating with a cobot on an assembly task and in particular to understand if gaze towards the cobot can serve as a natural cue to initiate joint action (**RQ1**). For this purpose, the Wizard of Oz experimental condition of the database collected by Mondellini et al. (2024), was selected. As shown in Figure 1, the wizard’s table was positioned on the opposite side of the cell with respect to the cobot working area so that if the operator’s gaze was directed towards the wizard, this behavior could be clearly identified and distinguished from a gaze towards their assembly table or towards the cobot itself. The role of the wizard was covered by one researcher, fluent both in Italian and English, able to monitor the system and to assist the operator during the task if needed.

#### 4.1.1 Participants and procedures

A total of 37 adult volunteers took part in the experiment (29 males and 8 females, all neurotypical) ranging from 18 to 48 years old (mean = 29.03, SD = 7.08). The participants where recruited (through personal connections or advertisements in public) among the employees of the institution or among students of a close-by University and they where all Italian except from 4 non-European volunteers. Prior to engaging in the assembly task, each participant was briefed about data treatment and signed a consent form (from which they can withdraw at any time) either in Italian or in English, depending on their preference. After that, appropriate training to the task was provided until the participant felt comfortable with the assembly steps to be performed (typically after a couple of assembly cycles). The experiment session duration of 15 min was carefully chosen to ensure an adequate number of assembly cycles (approximately 15–20 complete products) for each participant, enabling a comprehensive analysis of their recurring gaze behavior. With reference to Figure 2, each participant had to assemble Group B while the robot hovered with the detection camera over the pre-assembled Group A as if it was scanning for ready-to-pick sub-assemblies. As the volunteer’s task got close to completion, the wizard pressed a button on the laptop to trigger the robot. As a response, the robot smoothly interrupted the ongoing scanning motion, moved towards one of the sub-assemblies, picked it up, and brought it in front of the user at a convenient angle for the final joining, as shown in Figure 3. This iterative process continued throughout the 15-min experimental session, regardless of the number of completed gearboxes. To ensure a smooth workflow, ten pre-assembled sub-assemblies were initially placed on the cobot’s table. The researcher restocked the sub-assemblies as necessary. Importantly, participants were unaware of the trigger given by the researcher to prevent any potential biases in their behavior during the interaction with the cobot. Also, the participants were informed of being filmed for ethical reasons but the aim of studying their gaze behavior was revealed only at the end of the experiment, again to avoid any possible bias.

#### 4.1.2 Analysis

In human-human interactions, gaze-based social cues facilitate collaboration (Ferri et al., 2011; Innocenti et al., 2012). For example, the interaction is often initiated by looking at the other person. However, it is not known whether humans naturally exhibit similar gaze behavior when collaborating with an industrial cobot. To this end, we analyzed the gaze behavior of the participants working with a cobot on a collaborative assembly task. Specifically, we investigated if the participants gaze towards the cobot to initiate the collaborative joining of sub-assemblies. We note that the wizard controlled the cobot using the information about the completion of the sub-assembly and not their gazes. Hence, the participants were not required to exhibit any gaze pattern to complete the task. Moreover, they did not know what event triggered the cobot for joint activity. This setup allowed us to analyze the natural gaze behavior of the participants collaborating with a cobot, especially how they attempte to initiate the joint activity.

**FIGURE 7:**
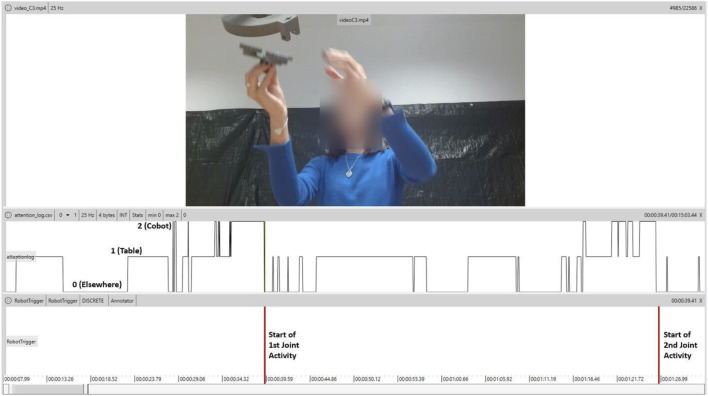
A snapshot from the NOVA tool showing the predictions from the attention recognition model (top track), and the annotated joint activity start points (bottom track, red lines).

We used an attention recognition model (see Section 3.2.1) to classify the gaze into three classes (0 - random, 1–table, 2–cobot). This model saves the annotation efforts involved in manually labeling the entire video. We used the NOVA tool (see Section 3.2.2) to annotate the frame where the cobot arrives for the collaborative joining of the sub-assemblies. This point was considered the start of the joint activity in each assembly cycle. In addition, we used NOVA to visualize the predictions from the attention recognition model along with the joint activity start points. With reference to Figure 7, the bottom track shows the annotated starting points of the joint activity. The values in the top track can be 0, 1, or 2 depending on the predicted class. We specifically focused on the instances where the predicted class is 2, i.e., the gaze is predicted towards the cobot. A promising trend was observed as spikes (class = 2) in the top track in the few seconds leading up to the joint activity. This pattern indicates that the participant was looking at the cobot plausibly to initiate the joint activity.

We analyzed this gaze pattern for each participant in two steps. First, we calculate the gazes to the cobot within 15 s prior to the joint activity. We chose 15 s because, after the trigger, the cobot takes 10–12 s to move over the part, grab it, pick it up, and bring it to the collaborative joining position (3 s). This step helped us determine how often the joint activity was preceded by gazing towards the cobot, and therefore a cue to initiate the activity. Second, we calculated the gazes to the cobot that were outside the above-mentioned 15 s and also outside the joint activity itself. This step allowed us to make sure that the gaze pattern was prominent around the time of the joint activity, and not a frequent behavior irrespective of the activity.

Before calculating the gazes, we smoothed the predictions from the attention recognition model using a three-point moving window. We used a peak detection algorithm to find the points where the gaze was directed towards the cobot. Again for smoothing reasons, we only considered the peaks that spanned for at least five frames (at 25 fps), i.e., the participant looked at the cobot for at least five consecutive frames. Using these peak points and the annotated starting points of the joint activity, we calculated the percentage of gaze-preceded joint activities (*pGazeJoint*) and the percentage of unexpected gazes to the cobot (*pUnexpectedGaze*). Joint activity was deemed gaze-preceded if the participant looked at the cobot at least once during the 15 s prior to the start of the joint activity. So, *pGazeJoint* (expressed in percentage) is the number of gaze-preceded joint activities out of the total joint activities in the session. We expected the participants to look at the cobot for initiating the joint activity and for the duration of the activity (typically lasts for 20–25 s). Any gaze towards the cobot that occurred outside this duration was considered unexpected. We calculated *pUnexpectedGaze* (expressed in percentage) as the ratio of unexpected gazes towards the cobot to the total number of gazes towards the cobot.

**FIGURE 8:**
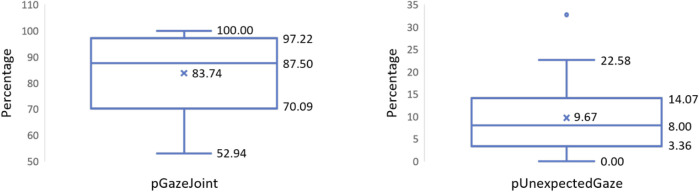
Box-plots computed from 37 participants representing *pGazeJoint* values on the left and *pUnexpectedGaze* values on the right.

Figure 8 visualizes the *pGazeJoint* and *pUnexpectedGaze* values from 37 participants as box-plots. The mean *pGazeJoint* value is 83.74, i.e., on average, 83.74% of all collaborative joining instances were preceded by a gaze towards the cobot. Similarly, the mean *pUnexpectedGaze* is 9.67%, which implies that only very few gazes at the cobot were outside the expected time frame. In other words, looking at the cobot occurs predominantly around the time of the collaborative joining activity. These results indicate that people use gaze as a social cue to initiate joint activity even when interacting with a cobot.

### 4.2 Experiment 2–Fully integrated system

With this second experiment, we wanted to pilot the full integration of the augmented collaborative cell where joint action is automatically triggered on the basis of the detected gaze behavior of the user. For this purpose, we used the same assembly task described for Experiment 1 but instead of having a Wizard triggering the joint action, we automated the process leveraging the attention recognition model presented in Section 3.2.1. In practice, the robot would automatically move towards the participant to perform the joint action only if the latter looked towards the robot for longer than a threshold tuned to avoid slowing down the collaboration flow but also to avoid unwanted activations due to quick glances. Thanks to this approach, Experiment 2 also offered a validation of the outcomes of Experiment 1 in terms of natural gaze behavior, following a logic inspired by Palinko et al. (2016) (**RQ2a**). Moreover, two comments collected from the participants of Experiment 1 led to additional research questions. Participant 3 stated that he thought the camera was involved in the synchronization mechanism of the system and therefore tried to look at it more often in order to speed up the task. Participant 34, instead, said that the noise of the robot scanning the parts was irritating and made it hard to focus on the task. Therefore, Experiment 2 was designed to also address two additional topics: to understand if the volunteers autonomously realize that their gaze is the source of automation for the system (**RQ2b**) and to explore if the robot’s scanning motion has any effect on the participants (**RQ2c**).

#### 4.2.1 Participants and procedure

With the mentioned goals in mind, two experimental conditions were designed and proposed to each participant in a randomized order. The first condition resembled almost completely Experiment 1, except for the fact that the trigger did not come from a wizard anymore, but was automatically generated on the basis of the user’s gaze behavior. The same automatic trigger was used also for the second condition but, in that case, the robot did not perform any hovering movement while waiting for the trigger but simply remained still over the pre-assembled components. With this approach, we wanted to understand if the results collected during Experiment 1 were somehow affected by the robot’s scanning motion. Also, we hypothesized that this second condition would make it easier for the participants to infer the role of their gaze in the task, since the robot would not do anything at all until the participant’s gaze was turned towards it.

A total of 10 volunteers were recruited for this second experiment. In terms of demographics, we had a balanced gender distribution (5 males and 5 females) and an age range going from 18 to 30 (mean = 23.8, SD = 5.14). All the participants were Italian and were mostly students of a close-by university. Moreover, nine of the participants were neurotypical while one of them was characterized by high-functioning Autism Spectrum Disorder (ASD). Interestingly, Mondellini et al. (2023) showed that some differences exist when comparing the behavior of neurotypical and ASD operators during a collaborative assembly task. Since the results collected from Experiment 1 were based on an entirely neurotypical experimental group, we decided to involve one ASD participant in order to explore the feasibility of the system outside the analyzed behavioral range. More ASD participants are planned to be involved in a similar experiment in the future and the new collected data will be part of a dedicated analysis. Similar to Experiment 1, participants were briefed about data treatment and signed a consent form from which they are free to withdraw at any moment. None of the participants had prior experience with the robot and they were not told about the gaze-based automatic triggering system. In order to keep the experiment as short as possible but still make sure to collect enough experience samples, we did not set a fixed duration for the sessions. Instead, each experimental condition lasted for the time required to assemble 10 complete gearboxes. A short break was provided between the two sessions to have the time to reset the system for the next condition. Finally, at the end of the second session, the participants were asked to report their impressions of the system, transcribed in the original language and then translated to English. Only after that, we briefed the participants about the automatic system and about the goals of the study.

The fully integrated system exploited the attention recognition model described in Section 3.2.1 to automatically trigger the cobot for joint activity, instead of the wizard. On top of that, we used the Social Signal Interpretation (SSI) (Wagner et al., 2013) framework, a Windows-based framework capable of recording, processing, and analyzing social signals. The input upper-body video frames were first cropped to the face region using MediaPipe’s face detection model called BlazeFace (Bazarevsky et al., 2019). Then, the attention recognition model was integrated inside an SSI pipeline in order to use the cropped face images as input to classify the gaze direction of the participant. After that, the classification results for each frame were sent to VSM (see Section 3.2.3) thanks to a specifically developed plugin designed to create a proper UDP connection with the SSI pipeline. Having this connection set up, the VSM program had to be slightly modified in order to produce the joint action trigger no longer on the basis of a keyboard press, as in Experiment 1, but using a specific logic based on the received attention recognition data (see Figures 5 and 6). In line with Experiment 1, we produced a valid trigger only if the participant was detected to be looking towards the robot for more than 5 frames. For this purpose, a counter was implemented inside the VSM program to keep count of the number of consequent frames of attention towards the cobot. Every time the user’s gaze was not detected to be directed towards the cobot, the counter was reset and the trigger was activated only if the counter exceeded the preset threshold of 5.

#### 4.2.2 Analysis

**FIGURE 9:**
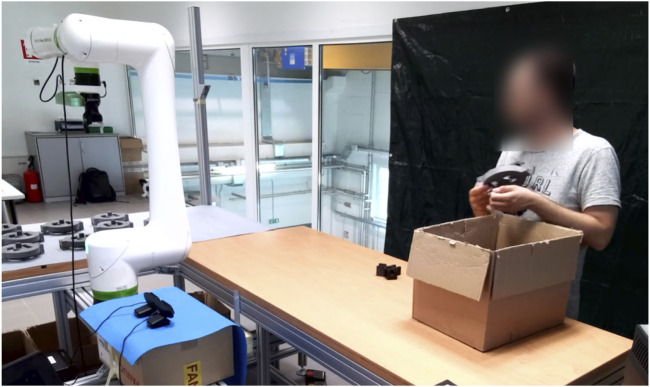
The ASD participant looking towards the cobot before starting to assemble his/her part of the gearbox.

The resulting fully integrated system was piloted with 10 volunteers who did not have prior experience with the robot and were not informed about the gaze-based triggering system. We considered a “successful interaction” every iteration in which the participant was able to trigger the joint action at the expected moment (right before/after finishing his/her part) and within a reasonable time (maximum of 5 s after finishing his/her sub-assembly, inspired by the threshold used by Eldardeer et al. (2020)). Once again, the NOVA tool (see Section 3.2.2) was used to annotate the frames corresponding to the moment when the participant was done with his/her part of the assembly and the moment when the cobot receives the trigger and starts moving towards its subassembly. Thanks to this annotation step, we were able to compute the amount of time passed between these two instances for each participant and for each assembly cycle. The value obtained for the first iteration at the start of every condition was excluded since it was affected by the start signal given by the researcher to the volunteer. The system achieved a success rate of 88.64% for the scanning condition, 94.38% for the condition with the robot standing still and an overall success rate of 91.53%. It is interesting to note that for all the iterations that were not considered successful, the participants actually looked at the robot and triggered the joint action but did that after the 5 s threshold set for the analysis. For both conditions, the system scored higher than what was observed during Experiment 1 (83.74% of joining instances preceded by a gaze towards the robot) meaning that full integration of the system can be considered successful. On average, during the scanning condition the participants had to wait 3.63 s after finishing their part to actually trigger the robot and see it start moving towards its subassembly. Considering the condition with the robot standing still, instead, the participants only had to wait for an average of 2.73 s, probably thanks to the time saved by not performing any scanning motion above the sub-assemblies. Moreover, some before-activations (i.e., the robot receiving the trigger before the end of the operator’s assembly task) were observed. Overall, this situation occurred 19.21% of the times with an average anticipation time of 2.19 s. A possible explanation for this result is that, over time, some of the volunteers may have guessed the role of their gaze in the process and started looking towards the robot before finishing their part in order to reduce the waiting times. A comparison between the average percentage of before activations of the group of participants who, at the end of the experiment, stated that they understood the gaze-based mechanism (before activations: 43.06%) and the others (before activations: 2.86%) seems to confirm the hypothesis. Interestingly, the average anticipation time also serves as a reference to highlight the unexpected behavior elicited by the ASD participant: instead of looking at the robot just before joint activity, s/he often looked towards it also before starting a new part, as clearly visible in Figure 9. As a result, the average anticipation time computed for this single volunteer is equal to 15.50 s, setting him/her apart from the rest of the experimental group. For this reason, the ASD participant has been treated an outlier and his/her data has been excluded from the computation of the quantitative measures.

## 5 Discussion

Our results show that people tend to look at the cobot when they are ready to work jointly on a task (RQ1) (represented by high *pGazeJoint*), a behavior prevalent in human-human interaction. This behavior can be seen as a social cue to initiate a joint activity, thereby promoting a more natural and intuitive human-robot collaboration. Additionally, our results indicate that gaze directed at the cobot typically occurs during the collaborative joining activity or shortly before the start of the joint activity, represented by low *pUnexpectedGaze*. We observed that longer joining times were one of the factors contributing to unexpected gazes towards the cobot. Specifically, during certain assembly cycles, participants took more time to align the sub-assemblies, resulting in a collaborative joining process that exceeded the estimated duration. Furthermore, errors or delays in the cobot’s performance were also responsible for unexpected gazes. For instance, in some cases, the robot did not initiate the subsequent assembly cycle immediately after completing the previous one due to unexpected software behaviors. Consequently, a few seconds of unforeseen delay preceded the next series of robot movements, capturing the participants’ attention and prompting them to look towards the robot to comprehend the situation.

As already mentioned, during Experiment 1 we also collected some insightful comments from the participants. Participant 3 said (translated from Italian): “I noticed that the robot was synchronized with me and I thought it might be because of the camera, so I tried looking at it to see what would happen”. Participant 37, instead, said (translated from Italian): “In some cases, I was surprised by how slow the robot was, so I tried looking at it in the hope of making it faster”. These participants inferred that their gaze influenced the cobot’s behavior; whereas in reality, during Experiment 1, it solely relied on the wizard’s judgment of whether the participant completed their sub-assembly. These comments further reinforce the idea of using gaze to facilitate more natural human-robot collaboration. Moreover, Participant 15 provided an interesting suggestion about adding eyes to the cobot to make it expressive. Although this suggestion relates to anthropomorphism and is beyond the scope of this work, it highlights a possible direction to make human-robot collaboration more natural.

Moving now to Experiment 2, the fully integrated system achieved an overall success rate of 91.53% demonstrating the feasibility of using the operator’s gaze information as a natural cue to trigger joint action with a cobot (RQ2a). In general, most of the participants reported a pleasant and natural interaction experience, again confirming the hypothesis of improving human-robot interaction patterns by leveraging the participant’s natural gaze behavior. An exception must be made for Participant 1 who stated (translated from Italian): “The noise and the waiting times of the robot were irritating”. Even though this aspect is not the focus of the present study, it is important to remember that the overall experience of a worker is the result of the combination of a variety of multi-sensory stimuli, which should all be taken into consideration to provide optimal working conditions.

As foreseen, most of the participants understood that something in their actions was triggering the robot to move for the joint action. A hint to this was already observed by the relevant percentage of before-activations observed during the experiment. Comparing the two sessions, often this feeling of having an effect on the behavior of the system was perceived more in relation to the condition where the scanning motion was absent. For instance, Participant 2 said (translated from Italian): “I think that during the scanning session, the robot had a fixed time before coming towards me. While in the still session, it came when I was done with my part.“. Again, this could be due to the fact that the scanning motion introduced a slight delay in the system response and therefore made it harder for the participants to intuitively connect their actions to the robot’s behavior. A total of four participants out of ten correctly identified their gaze as the source of automation (RQ2b). The others either thought that the robot was going through a fixed schedule or that it was triggered by some other features such as their body position or their action of lifting the sub-assembly from the table.

Comparing the two experimental conditions, most of the participants preferred the one without the scanning motion because they perceived the robot as more reactive and better synchronized to their actions. This result is confirmed by the computed average waiting time and can be easily explained by the fact that in the case where the robot remained still waiting for the trigger, the motion towards the sub-assembly started as soon as the trigger was received. On the other hand, during the scanning condition, as soon as the trigger was generated the robot had to smoothly interrupt the hovering motion and only then move towards the part, therefore adding a small delay in the actual start of the joint action. However, as stated, all the participants were able to successfully interact with the robot in both cases, therefore ruling out the existence of an effect of said scanning motion over the natural gaze behavior of the participants during the task (RQ2c).

A noticeable difference was observed when piloting the system with a participant characterized by ASD: in most of the cases, the robot got triggered much earlier and ended up waiting for the user before joint action. Even though this behavior was observed during both experimental conditions, it happened more frequently when the robot was not performing the scanning motion. As a first hypothesis, the stillness of the robot may have been unconsciously perceived by the participant as a fault of the system, attracting his/her attention to make sure everything is under control. Of course, as soon as the ASD participant directed his/her attention towards the robot, the trigger was produced, the robot started moving and the participant went back to focusing on his/her part of the assembly. Surprisingly, when asked about his/her experience during the two sessions, the participant revealed that s/he had not noticed any difference in the robot’s behavior (Translated from Italian: “I felt smooth working with the robot during both conditions”). Even though the participant did not express any discomfort related to the unexpected triggers and the task was nevertheless carried out without any issues, this result highlights how different groups of individuals may have different needs and elicit different behavioral patterns which should be taken into account when designing human-robot collaboration strategies.

### 5.1 Conclusions and future works

As the literature suggests, the collaboration experience of cobot workers can be improved by incorporating elements from human-human interactions. In this work, we performed two experiments aiming to investigate if people’s gaze behavior can successfully be used as a natural cue to initiate joint activity. Although this behavior is common in human-human interactions, it is not known if such behavior occurs during human-robot collaborations.

To this end, we designed Experiment 1 to study the gaze behaviors of 37 participants collaborating with a cobot in an industry-like assembly task. We used a Wizard of Oz setup to trigger the collaborative joining activity. Using a gaze-based attention recognition model, we identified the instances where the participant looked at the cobot. Our analysis revealed that 83.74% of the joint activities were preceded by a gaze towards the cobot. We also found that, in the entire assembly cycle, the participants tended to look at the cobot around the time of the joint activity. Our results indicate that the gaze-based initiation cue indeed extends to human-robot collaboration.

Hence, we designed Experiment 2 in order to pilot the fully integrated system with 10 participants, generally achieving smooth and natural interaction experiences and an overall success rate of 91.53%. Interestingly, we notice relevant differences in the interaction with the system between neurotypical participants and the participant with ASD, highlighting the need for further investigations to understand how such a system could be adapted to respond in a natural way also to users diverging from the neurotypical behavior.

In the future, we will study if the gaze-based initiation cue is valid in longer collaboration sessions, with a larger sample size and in a real-life setting (e.g., actual industrial workcell). For instance, the participants may start expecting the cobot to know the appropriate time for joint activity, even without any cues from the participant. Also, we want to explore the differences in terms of natural gaze behavior between neurotypical participants and participants characterized by ASD. Since a mix of Italian and English speaking participants took part in the study, it would be interesting to further analyze if the translated instructions had had any effect on the results, although unlikely in the author’s opinion since the explanation was paired with a practical training on the task. Another point of interest would be the augmentation of the presented gaze-based triggering system with action recognition functionalities to ensure that unexpected situations are dealt correctly by the robot. Lastly, we will explore and try to quantify the benefits of the fully integrated adaptive behavior of the cobot in terms of the wellbeing and experience of the operators. In doing so we will transition the software architecture to the latest ROS2 LTS distribution in order to guarantee state-of-the-art performance for the system.

## Data Availability

The raw data supporting the conclusions of this article will be made available by the authors, without undue reservation.
